# An evaluation of the psychometric properties of the sf-12v2 health survey among adults with hemophilia

**DOI:** 10.1186/s12955-018-1059-8

**Published:** 2018-12-13

**Authors:** Ruchitbhai M. Shah, Benjamin F. Banahan, Erin R. Holmes, Amit S. Patel, Marie Barnard, Rahul Khanna, John P. Bentley

**Affiliations:** 1 Pharmerit International, 4350 East West Hwy #1110, Bethesda, MD 20814 USA; 2 Department of Pharmacy Administration, University of Mississippi, University, USA; 3 Medical Marketing Economics, Oxford, USA; 4 Johnson & Johnson, New Brunswick, USA

## Abstract

**Background:**

This study examined the psychometric properties of version 2 of the SF-12 Health Survey (SF-12v2) among adults with hemophilia in the United States.

**Methods:**

This study employed a cross-sectional design using web-based and paper-based self-administered surveys. Hemophilia patients were recruited using an online panel and at a hemophilia treatment clinic. The psychometric properties of the SF-12v2 were assessed in terms of construct validity, internal consistency reliability, and presence of floor and ceiling effects.

**Results:**

A total of 218 adults with hemophilia completed the survey, with most recruited via the online panel (78%). Confirmatory factor analysis using the WLSMV estimator in Mplus supported a two-factor model for the SF-12v2 where the physical functioning, role physical, bodily pain, and general health items loaded onto a latent physical factor (LPF) and the role emotional, mental health, social functioning, and vitality items loaded onto a latent mental factor (LMF). Model fit statistics for the two-factor model were: Chi-square [df] = 172.778 [48]; CFI = 0.972; TLI = 0.962; RMSEA [90% CI] = 0.109 [0.092–0.127]; WRMR = 0.947. Correlated residuals for items belonging to similar domains were estimated and there was a significant correlation between LPF and LMF. All standardized factor loadings were strong and statistically significant, indicating adequate convergent validity. Item-to-other scale correlations were lower than item-to-hypothesized scale correlations suggesting good item discriminant validity. Model testing revealed that LPF and LMF were not perfectly correlated, suggesting adequate construct discriminant validity. Increasing levels of symptom severity were associated with significant decreases in physical component summary (PCS) and mental component summary (MCS) scores, supporting known-groups validity. Internal consistency reliability was satisfactory, with Cronbach’s alpha of 0.848 for the LPF and 0.785 for the LMF items. Finally, none of the participants received the least or maximum possible PCS or MCS score, indicating the absence of floor and ceiling effects.

**Conclusions:**

Overall, the SF-12v2 was found to have adequate psychometric validity in our sample of adults with hemophilia. These results add to the growing evidence of psychometric validity of the SF-12v2 in different patient populations including hemophilia.

## Background

Hemophilia is a rare X-linked chronic genetic blood coagulation disorder seen predominantly among males. It is caused by a deficiency of clotting factors VIII or IX in blood plasma. It affects about 400,000 people across the world and about 20,000 in the United States (US) [1, 2]. Patients with hemophilia experience bleeding into joints and muscles which in severe cases can lead to chronic pain, reduce the range of joint motion and eventually progress to chronic arthritis [3].

For patients living with hemophilia, merely treating and preventing bleeding episodes and other physical symptoms using clotting factor concentrates is not enough. Patients with hemophilia must be careful about participating in activities such as contact sports because immediate bleeding may ensue. Long-term impairments in mobility and impact on functional status due to reduced range of joint motion may also limit the activities in which patients can participate. This can affect social participation and peer integration [4, 5]. Employment and occupational disabilities can occur as well. Also, the disease can influence the mental well-being of patients within whom signs of depression, anxiety and psychological distress are common [6]. Thus, the physical, mental and social consequences of the disease serve to reduce the HRQOL of patients. Therefore, HRQOL assessment is now recognized as an important health outcomes endpoint which can help decide and optimize treatment options among patients with hemophilia. Overall HRQOL is a multidimensional, subjective concept which incorporates physical functioning, psychological functioning, social interaction, and somatic sensation [7].

One key aspect of measuring HRQOL of a population is the selection of the appropriate instrument. The SF-12 Health Survey version 2 (SF-12v2) is a generic measure of HRQOL [8]. Generic instruments allow for comparison of patients’ health status across disease states and conditions [9]. Generic HRQOL measures may be less sensitive to certain key aspects or symptoms of a particular disease state and as a result may not be able to capture small changes in the HRQOL of patients having a certain disease [10]. On the other hand, disease-specific HRQOL measures focus on problems that may be specific to a disease population. However, these instruments cannot be used to compare HRQOL across different disease states. Such information may be important to clinicians and policy makers in making key treatment and resource allocation decisions. Given their underlying utility, it is necessary to obtain evidence about the appropriateness of use of generic HRQOL measures (such as the SF-12v2) in different patient population [11].

Initial evidence regarding the reliability and validity of the SF-12 in the general US population was provided by Ware and colleagues in 1996 using data from the National Survey of Functional Health Status (NSFHS) and the Medical Outcomes Study (MOS) [12]. The instrument has since been evaluated for use among general populations in several different countries such as Denmark, Germany, United Kingdom, Netherlands, United States and others [13–16] as well as among patients with different diseases including Parkinson’s disease, stroke, diabetes mellitus, inflammatory rheumatic disease, hemodialysis [17–21]. The results of these studies suggest that the SF-12 has good psychometric properties. The SF-12v2 is an abbreviated version of the SF-36, which is one of the most commonly used generic HRQOL measures [9].

Although the SF-12v2 has been used to assess the HRQOL of hemophilia patients [22, 23], its psychometric properties have never been established among patients with hemophilia. To ascertain that the SF-12v2 is appropriate for use among hemophilia patients, its psychometric properties must be established in this population. Therefore, this study evaluated the psychometric properties of the SF-12v2 among adult patients with hemophilia in the US. The psychometric properties of the SF-12v2 assessed included: convergent validity, discriminant validity, known-groups validity, factorial validity using confirmatory factor analysis, and internal consistency reliability. Presence of floor and ceiling effects was also examined.

## Methods

### Setting

A cross-sectional design using a web-based, self-administered survey was distributed to a national convenience sample of adults with hemophilia in the United States. Study approval was obtained from the University of Mississippi Institutional Review Board under the exempt status.

Potential participants were sent an email explaining the objective and scope of the study. This email assured the respondents that their information would be kept confidential. The email also contained a URL link to the survey which was programmed in Qualtrics [24]. The survey was open from October 31, 2015 to January 31, 2016. All respondents were provided $10 Amazon gift cards for participation in the study.

### Participants

The sample included adults (≥ 18 years of age) with hemophilia A or B. Patients with other blood coagulation disorders such as Von Willebrand’s disease were excluded from the study sample. The sample was recruited with the help of a market research vendor company called Rare Patient Voice [25] which maintains a panel of hemophilia patients who were primarily recruited at hemophilia-related conferences and patient advocacy group meetings across the US. Considering hemophilia is a rare disease, patients were also recruited using a Facebook community of hemophilia patients called Hemo Friends and at the University of Mississippi (UMMC) hemophilia treatment center (HTC) to maximize the analyzable sample size for the current study. In this study, 169 (77.5%) patients were recruited using the Rare Patient Voice panel, 44 (20.2%) from the Hemo Friends Facebook community, and 6 (2.3%) from UMMC. Given the nature of the statistical analysis plan for this study (i.e., confirmatory factor analysis), an a priori sample size of 200 patients with hemophilia was considered to be adequate [26].

### Instruments

Patients with hemophilia were asked to describe their HRQOL using the SF-12 Health Survey Version 2 (SF-12v2). The SF-12 is the shorter version of the SF-36 [12]. The SF-12v2 is a generic health profile instrument with 12-items which compose 8 health concepts forming a health profile [8]. These eight sub-domains are: physical functioning (PF), role physical (RP), bodily pain (BP), general health (GH), vitality (VT), social functioning (SF), role-emotional (RE), and mental health (MH). These eight sub-domain scores can be weighted and summarized into two component scores – the physical component summary (PCS) score and the mental component summary (MCS) score. According to the theoretical test model, the items from the physical functioning, role-physical, bodily pain, and general health sub-domains are primarily indicators of PCS while vitality, social functioning, role-emotional, and mental health items are primarily indicators for MCS [18]. For the SF-12v2, the norm-based PCS and MCS scores for the general US population have a mean of 50 and a standard deviation of 10 with higher scores indicating a better health status [8]. PCS, MCS, and sub-domain scores were calculated using the scoring software available from Optum (using 2009 US norms).

Hemophilia-related symptom severity was reported using the Patient Global Impression of Severity (PGI-S). The PGI-S is a single self-reported item that asks respondents to rate the severity of their disease condition. In this study, the PGI-S was worded: “When thinking about all of the hemophilia-related symptoms that you may have experienced during the past 4 weeks, please indicate the one option that best describes how your symptoms overall have been: (1) no symptoms, (2) mild symptoms, (3) moderate symptoms, or (4) severe symptoms.” A similar self-reported symptom severity measure has been used in studies of males with lower urinary tract symptoms secondary to benign prostatic hyperplasia and women with stress urinary incontinence [27, 28].

### Analysis

A descriptive analysis of the individual SF-12v2 items was conducted in terms of means and standard deviations (SD). Missing data, if any, was reported in terms of frequencies and percentages on a per-item level. Kurtosis and skewness coefficients were also calculated and variables with absolute value of the skew index > 3.0 and kurtosis index > 10.0 were considered to be non-normal [26]. Descriptive statistics were calculated for all other study variable in the form of frequencies and percentages for categorical variables, means and standard deviations for continuous variables.

Confirmatory factor analysis (CFA) was used to evaluate the factor structure of the SF-12v2 among patients with hemophilia. CFA is a structural equation modeling technique which can be used to evaluate the fit of a theoretically-based measurement model. Three measurement models were tested. First, a 1-factor model (Model 1) which forced all the SF-12v2 items to load on a single latent factor. Second, a 2-factor model based on the approach adopted by Okonkwo and colleagues (Model 2) [18] where the PF, RP, and BP items were specified to load on a latent physical health factor (LPF), the RE and MH items were specified to load on a latent mental health factor (LMF), and the three GH, VT, and SF items were allowed to load on both latent factors. The residuals for the two PF items were allowed to correlate in both the 1-factor and 2-factor models as modeled by Okonkwo et al. [18]. Third, a 2-factor model employed by Maurischat and colleagues (Model 3) [20] where the GH, PF, RP, and BP items were specified to load on a LPF and the RE, MH, VT, and SF items were allowed to load onto a LMF. The residuals for each of the two PF, RP, RE, and MH items were allowed to correlate as modeled by Maurischat et al. [20]. In both models 2 and 3, LPF and LMF were allowed to correlate.

Considering that the items on the SF-12v2 are ordered categorical variables with limited response options along with the possibility of item responses that skewed toward one end (i.e., the presence of floor or ceiling effects), weighted least squares estimation (WLSMV) for categorical indicators was used to quantify the hypothesized relationships [29]. All CFA models were estimated using Mplus version 7.31 (Muthen & Muthen, Los Angeles, CA). Model fit for each model was assessed using the following five fit statistics: χ^2^ statistic, the root mean square error of approximation (RMSEA), the Tucker Lewis Index (TLI), the comparative fit index (CFI), and the weighted root mean square residual (WRMR). Bagozzi and Yi (2012) suggest that for a well-fitting model, the RMSEA, TLI, CFI must be ≤0.08, ≥0.92, and ≥ 0.93 respectively [30]. For a good fitting model, WRMR must be less than or equal to 1 [31].

Given that the LPF and LMF loadings are sample specific, an additional model was fit to estimate the correlations of the latent factors with PCS and MCS scores from the standard algorithm.

Factor loadings from the CFA models and item-scale correlations were used to assess convergent validity among the items. The size of the factor loading is an indication of the amount of variance in a particular item that is explained by the latent construct. For the current study, standardized factor loadings that were statistically significant and greater than 0.5 were considered to be indicative of good convergent validity [26, 31]. Statistical significance of the factor loadings was considered as a minimum requirement because a significant loading could be weak or moderate in strength.

Higher item-scale correlations (Pearson’s correlation between score on an individual item in a sub-domain with the total score on the underlying sub-domain) indicate that expected items in the same sub-domains correlate strongly with each other. This approach of establishing convergent validity has been used by previous studies [32]. Item-scale correlations of 0.1–0.29 were considered small, 0.3–0.49 as moderate, and ≥ 0.45 was considered to be suggestive of strong [33]. A strong correlation of the items belonging to the GH, PF, RP, and BP sub-domains with PCS was hypothesized. Similarly, a strong correlation between items representing the RE, MH, VT, SF sub-domains with MCS was hypothesized.

To assess latent construct discriminant validity, the fit of the best fitting 2-factor model obtained from the factorial validity analysis was compared to that of a similar model where the latent factor correlation (i.e., correlation between LPF and LMF) was fixed to 1; this test was carried out using the DIFFTEST option in Mplus [34, 35]. A significant difference in the model fit (χ^2^ statistic) between the two models was suggestive of discriminant validity [36].

To assess item discriminant validity [37], lower item-to-other scale correlations (≤ 0.40) were suggestive of adequate discriminant validity. The reasoning behind this technique was that items from different domains should have low or no correlations with each other. A weak correlation of the items belonging to the GH, PF, RP, and BP sub-domains with MCS was hypothesized. Similarly, a weak correlation between items representing the RE, MH, VT, SF sub-domains with PCS summary scale score was hypothesized.

Known-groups validity is the ability of an instrument to differentiate among individuals who have varying levels of disease severity. One-way ANOVA was used to compare mean PCS and MCS scores from the SF-12v2 across hemophilia patients with different symptom severity levels measured using the PGI-S.

In order to evaluate the internal consistency reliability for the SF-12v2, Cronbach’s alpha (α) was calculated for the LPF and the LMF items. An α ≥ 0.70 was considered to be suggestive of adequate internal consistency reliability [32].

To assess the floor and ceiling effects of the SF-12v2, the percentage of adults with hemophilia with the least possible and the maximum possible PCS and MCS were determined. Floor and ceiling effects were considered to be present if more than 20% of the respondents received the lowest or the highest possible PCS or MCS score [32, 38]. Given the estimation technique used for the CFA and the treatment of the items as categorical indicators, floor and ceiling effects of the individual SF-12v2 items were not considered to be problematic.

## Results

The final study sample consisted of 218 adults with hemophilia (Table 1). The majority of the sample included patients with hemophilia A (77.5%), males (79.5%), and Caucasians (68.5%). The mean age of the study sample was 35.45 (12.3) years. Hepatitis C (36.5%) and depression (38.4%) were the most commonly reported comorbidities.

**Table 1:** Demographic and clinical characteristics of the sample

Characteristics	*N* (%)
Diagnosis	
Hemophilia A	169 (77.5)
Hemophilia B	49 (22.5)
Gender	
Male	174 (79.5)
Female	24 (11.0)
Age, mean (sd)	35.45 (12.3)
Race/Ethnicity	
White/Caucasian	150 (68.5)
Other^a^	47 (21.5)
Marital Status	
Never Married	66 (30.1)
Married	98 (44.7)
Other^b^	34 (20.1)
Education level	
Less than high school	9 (4.1)
High school or technical school	30 (13.7)
College degree	128 (58.4)
Masters degree	17 (7.8)
Doctoral degree	6 (2.7)
Professional degree	8 (3.7)
Employment Status	
Employed/self–employed full time	103 (47.0)
Employed part–time	23 (10.5)
On disability	16 (7.3)
Other^c^	56 (25.6)
Region of the country	
Northeast	46 (21.0)
Midwest	40 (18.3)
South	54 (24.7)
West	58 (26.5)
Health Insurance	
Public	61 (27.9)
Private	113 (51.6)
Both	6 (2.7)
None	18 (8.2)
Symptom Severity^e^	
No symptoms	31 (14.2)
Mild symptoms	67 (30.6)
Moderate symptoms	61 (27.9)
Severe symptoms	39 (17.8)
Treatment Regimen	
Always received on prophylaxis therapy	121 (55.3)
Received both prophylaxis and on-demand therapy	31 (14.2)
Always received on on-demand therapy	45 (20.5)
Number of bleeding episodes in the past year, mean (sd)	11.69 (19.1)
Hemophilia-related surgery	
Yes	73 (33.3)
No	124 (56.6)
Comorbidities	
Hepatitis C	80 (36.5)
HIV^d^	26 (11.9)
Depression	84 (38.4)
Inhibitors to clotting factor	41 (18.7)
Annual number of hemophilia-related visits, mean (sd)	
Hemophilia Treatment Center (HTC)	2.23 (3.1)
Hematologist outside a HTC	0.83 (1.8)
Primary Care Practitioner	2.42 (4.2)

Note: SD, standard deviation; Frequencies for each demographic characteristic may not add up to the total sample size due to the presence of missing data

^a^Other includes American Indian/Alaskan Native, Asian/Indian Asian, Native Hawaiian/Other Pacific Islander, Hispanic, etc.

^b^Other includes divorced, separated, widowed, and not married, living with partner

^c^Other includes retired, home–maker, student, seeking work, etc.

^d^*HIV* Human Immunodeficiency Virus

^e^Based on Patient Global Impression of Severity (PGI-S)

Table 2 shows the mean scores, and skewness and kurtosis coefficients on a per-item basis for the SF-12v2. The skewness and kurtosis coefficients for all items on the SF-12v2 were found to be within a range of − 1.00 and 0.63. The mean PCS was 43.68 (SD = 10.20) and the mean MCS was found to be 46.48 (SD = 10.09) among adults with hemophilia. Mean PCS and MCS scores were lower than the norm scores for the general healthy US population. This indicated that adults with hemophilia had a worse overall HRQOL as compared to the US norm population. There was no missing data for any of the SF-12v2 items.

**Table 2:** SF-12v2 item-level characteristics and PCS/MCS scores among adults with hemophilia

Items	N	Missing	Mean (SD)	Skewness		Kurtosis	
				Statistic	Std. error	Statistic	Std. error
Health in general (GH01)	218	0	2.89 (0.94)	0.28	0.164	−0.22	0.327
Limitation in moderate activities (PF02)	218	0	2.25 (0.68)	−0.36	0.164	−0.86	0.327
Limitation in climbing stairs (PF03)	218	0	2.17 (0.78)	−0.3	0.164	−1.00	0.327
Accomplished less due to physical health (RP04)	218	0	3.32 (1.27)	−0.24	0.164	−0.88	0.327
Limited in health due to physical health (RP05)	218	0	3.25 (1.26)	−0.12	0.164	−0.96	0.327
Pain interfered with work (BP08)	218	0	2.91 (1.18)	0.09	0.164	−0.94	0.327
Have a lot of energy (VT10)	218	0	3.09 (1.03)	0.1	0.164	−0.50	0.327
Physical health or emotional problems interfered with social activities (SF12)	218	0	3.57 (1.12)	−0.43	0.164	−0.42	0.327
Accomplished less due to emotional problems (RE06)	218	0	3.55 (1.29)	−0.46	0.164	−0.95	0.327
Less careful due to emotional problems (RE07)	218	0	3.80 (1.20)	−0.63	0.164	−0.62	0.327
Felt calm and peaceful (MH09)	218	0	2.62 (0.98)	0.42	0.164	−0.36	0.327
Felt downhearted and depressed (MH11)	218	0	3.65 (1.10)	−0.55	0.164	−0.47	0.327
PCS score	218	0	43.68 (10.20)	−0.62	0.164	0.61	0.327
MCS score	218	0	46.48 (10.09)	−0.72	0.165	1.23	0.328

Note: Responses to the items on the SF-12v2 were measured using 3-point (PF02 and PF03) or 5-point (GH01, RP04, RP05, RE06, RE07, BP08, MH09, VT10, MH11, SF12) response scales

Note: GH01, BP08, MH09, and VT10 are SF-12v2 items where higher scores indicate poorer health status; all other items are scored such that higher scores indicate better health status

**Fig. 1:**
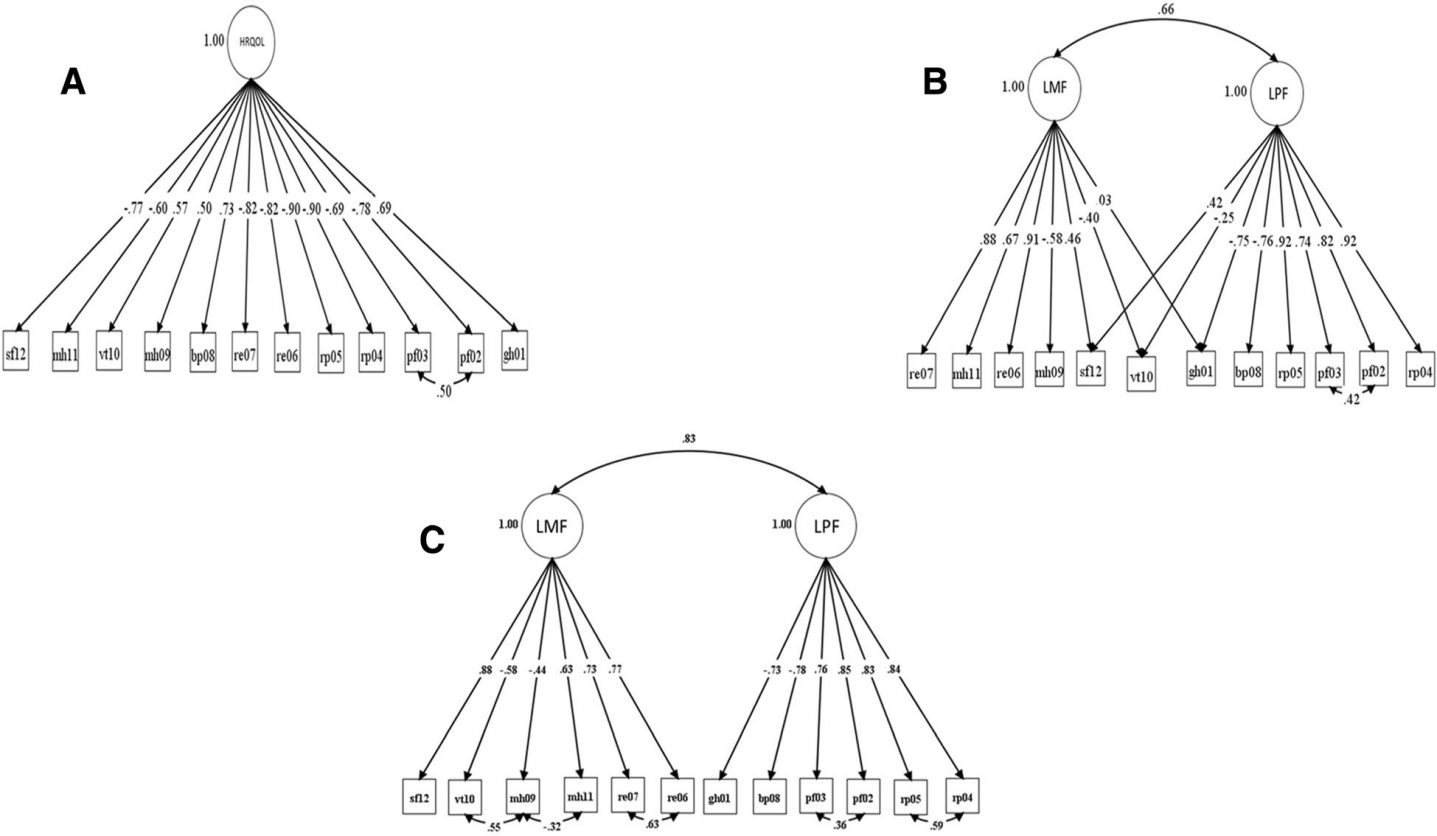
**a**: Single-Factor Model (Model 1) for the SF-12v2. **b**: Two-Factor Model (Model 2) for the SF-12v2 based on Okonkwo et al. **c**: Two-Factor Model (Model 3) for the SF-12v2 based on Maurischat et al.

The three measurement models tested to examine the factorial validity of the SF-12v2 among adults with hemophilia can be found in Fig. 1. The model fit indices for the three models can be found in Table 3. The two-factor model based on the approach used by Maurischat et al. [20] had the best fit among the three models (Chi-square [df] = 270.183 [49]; CFI = 0.952; TLI = 0.935; RMSEA [90% CI] = 0.144 [0.127–0.162]; WRMR = 1.250). Based on the modification indices for Model 3, residuals for items 9 and 10 (i.e., MH09 and VT10) were correlated in addition to the correlated residuals already specified in the Maurischat et al. model. This significantly improved model fit of the final model (Chi-square [df] = 172.778 [48]; CFI = 0.972; TLI = 0.962; RMSEA [90% CI] = 0.109 [0.092–0.127]; WRMR = 0.947).

**Table 3:** Summary of model fit indices for the SF-12v2 confirmatory factor models

Fit Statistics	Model 1	Model 2	Model 3
Chi-square (*df*)	531.807 (53)	340.418 (49)	172.778 (48)
CFI	0.896	0.936	0.972
TLI	0.870	0.914	0.962
RMSEA (90% *CI*)	0.203 (0.188–0.219)	0.165 (0.149–0.182)	0.109 (0.092–0.127)
WRMR	2.011	1.409	0.947

Model 1 - All items load onto a single latent health-related quality of life factor

Model 2 - SF-12 CFA model based on Okonkwo et al.

Model 3 - SF-12 CFA Model based on Maurischat et al.

Note: *df* degrees of freedom, *CFI* Comparative Fit Index, *TLI* Tucker-Lewis Index, *RMSEA* Root Mean Square Error of Approximation, *WRMR* Weighted Root Mean Square Residual, *CI* Confidence Interval

For the best fitting model (i.e., Model 3 in Fig. 1c), the correlation between LPF and the PCS score was 0.996 (*p* < 0.0001) while the correlation between LMF and MCS was > 0.999 (*p* < 0.0001). This suggested that there was a high and significant correlation between the sample-specific latent factors (i.e., LPF and LMF) and the PCS and MCS scores calculated using population-based weighting coefficients.

The standardized factor loadings for the final study model (Fig. 1c) can be found in Table 4. All factor loadings were statistically significant (*p* < 0.05). Most factor loadings (except MH09 on LMF) were greater than 0.5. Table 5 depicts the item-scale correlation matrix. Items comprising the PF, RP, GH, and BP sub-domains had a strong and statistically significant correlation with the PCS. While RE, MH, VT, SF items were strongly correlated with the MCS summary scale score. Overall, the standardized factor loadings and item-scale correlations suggested acceptable convergent validity for the SF-12v2 among adults with hemophilia.

**Table 4:** Standardized factor loadings for the final two-factor model of HRQOL (Model 3) for the SF-12v2 among adults with hemophilia

Items	Estimateˠ (SE)
Latent Physical Factor	
Health in general (GH01)	−0.734 (0.040)
Limitation in moderate activities (PF02)	0.848 (0.028)
Limitation in climbing stairs (PF03)	0.758 (0.038)
Accomplished less due to physical health (RP04)	0.842 (0.025)
Limited in health due to physical health (RP05)	0.831 (0.026)
Pain interfered with work (BP08)	−0.785 (0.030)
Latent Mental Factor	
Accomplished less due to emotional problems (RE06)	0.766 (0.044)
Less careful due to emotional problems (RE07)	0.726 (0.037)
Felt calm and peaceful (MH09)	−0.443 (0.054)
Have a lot of energy (VT10)	−0.575 (0.048)
Felt downhearted and depressed (MH11)	0.627 (0.040)
Physical health or emotional problems interfered with social activities (SF12)	0.878 (0.028)
Latent Factor Correlation	
Latent Physical Factor with Latent Mental Factor	0.829 (0.039)
Correlated Residuals	
Item PF02 with PF03	0.355 (0.091)
Item RP04 with RP05	0.591 (0.047)
Item RE06 with RE07	0.627 (0.041)
Item MH09 with VT10	0.552 (0.054)
Item MH09 with MH 11	−0.323 (0.049)

Items with negative factor loadings represent those SF-12v2 questions on which higher scores indicated poorer health status whereas items with positive factor loadings represent the SF-12v2 questions on which higher scores reflected better health status

ˠ All factor loadings were significant at α = 0.05

**Table 5:** Item-scale correlations for the SF-12v2 among adults with hemophilia

	GH	PF	RP	RE	BP	MH	VT	SF	PCS	MCS
GH										
GH01	−.990^**^	−.592^**^	−.575^**^	−.292^**^	−.455^**^	−.323^**^	−.502^**^	−.418^**^	−.737^**^	−.266^**^
PF										
PF02	.525^**^	.892^**^	.651^**^	.469^**^	.555^**^	.335^**^	.339^**^	.588^**^	.759^**^	.282^**^
PF03	.540^**^	.918^**^	.587^**^	.291^**^	.514^**^	.285^**^	.338^**^	.444^**^	.791^**^	.137^*^
RP										
RP04	.541^**^	.642^**^	.955^**^	.554^**^	.600^**^	.315^**^	.302^**^	.554^**^	.761^**^	.321^**^
RP05	.543^**^	.659^**^	.955^**^	.522^**^	.575^**^	.315^**^	.327^**^	.539^**^	.769^**^	.304^**^
RE										
RE06	.258^**^	.383^**^	.562^**^	.945^**^	.431^**^	.472^**^	.300^**^	.584^**^	.258^**^	.742^**^
RE07	.275^**^	.394^**^	.495^**^	.936^**^	.457^**^	.450^**^	.287^**^	.546^**^	.258^**^	.717^**^
BP										
BP08	−.454^**^	−.588^**^	−.615^**^	−.472^**^	− 1.00^**^	−.376^**^	−.391^**^	−.577^**^	−.736^**^	−.350^**^
MH										
MH09	−.364^**^	−.261^**^	−.210^**^	−.285^**^	−.342^**^	−.834^**^	−.599^**^	−.376^**^	−.159^*^	−.671^**^
MH11	.173^*^	.318^**^	.346^**^	.538^**^	.303^**^	.872^**^	.313^**^	.490^**^	.095	.761^**^
VT										
VT10	−.485^**^	−.374^**^	−.329^**^	−.312^**^	−.391^**^	−.524^**^	− 1.00^**^	−.425^**^	−.371^**^	−.592^**^
SF										
SF12	.414^**^	.554^**^	.573^**^	.602^**^	.577^**^	.511^**^	.425^**^	1.00^**^	.494^**^	.662^**^

Note: The following sub-domain scores were computed - *GH* general health, *PF* physical functioning, *RP* role physical, *VT* vitality, *SF* social functioning, *RE* role emotional, *MH* mental health

The following summary scores were computed - PCS, Physical Component Summary; MCS, Mental Component Summary

Note: GH01, BP08, MH09, and VT10 are SF-12v2 items where higher scores indicate poorer health status; all other items are scored such that higher scores indicate better health status

**Correlation is significant at the 0.01 level

*Correlation is significant at the 0.05 level

The fit of the final two-factor model (Model 3) where the correlation between LPF and LMF was freely estimated was compared to that of a model where the correlation between LPF and LMF was fixed to one. Although the correlation between LMF and LPF was high (*r* = 0.83), the test yielded a significant difference in the chi-square value (Δχ^2^ [df] = 18.686 [1]; *p* < 0.0001), suggesting that LPF and LMF are not perfectly correlated (i.e., latent construct discriminant validity) [34, 36]. Items comprising the PF, RP, GH, and BP subdomains had a weak correlation with the MCS summary scale score. While RE, MH, VT, SF items had a weak to moderate correlation with the PCS summary scale score, supporting item discriminant validity. Overall the SF-12v2 was found to have acceptable discriminant validity among adults with hemophilia.

The ability of the SF-12v2 to discriminate among hemophilia patient groups defined by the PGI-S (i.e., no symptoms, mild symptoms, moderate symptoms, and severe symptoms) was assessed using a one-way ANOVA (Table 6). Differences in PCS and MCS scores between individual groups were assessed using Tukey’s honestly significant difference (HSD) tests. The mean PCS (50.10 vs 47.39 vs 40.79 vs 35.24; *p* < 0.0001) and MCS (50.61 vs 46.80 vs 46.96 vs 42.29; *p* = 0.007) scores were significantly different across the four symptom severity levels. A definite gradation was observed in terms of PCS and MCS mean scores with increasing levels of symptom severity on the PGI-S.

**Table 6:** Known-groups validity for the SF-12v2 components among adults with hemophilia

Component	Group 1 No symptoms (*N* = 31)	Group 2 Mild Symptoms (*N* = 67)	Group 3 Moderate Symptoms (*N* = 61)	Group 4 Severe Symptoms (*N* = 39)	
	Mean (SD)	Mean (SD)	Mean (SD)	Mean (SD)	*p-*value
Physical Component Summary Score (PCS)	50.10 (7.92)^ˠ^	47.39 (8.25)^*^	40.79 (8.59)^ˠ*¥^	35.24 (10.78)^ˠ*¥^	< 0.0001
Mental Component Summary Score (MCS)	50.61 (8.23)^√^	46.80 (9.53)	46.96 (9.27)	42.29 (12.28)^√^	0.007

ˠSignificant difference in mean PCS scores between group 1 as compared to group 3 and group 4 (*p* < 0.05) based on Tukey’s HSD (Honestly Significant Difference) test

*Significant difference in PCS scores between group 2 as compared to group 3 and group (*p* < 0.05) based on Tukey’s HSD test

^¥^Significant difference in PCS scores between group 3 and group 4 (*p* < 0.05) based on Tukey’s HSD test

^√^Significant difference in mean MCS scores between group 1 and group 4 (*p* < 0.05) based on Tukey’s HSD test

Symptom severity categories are based on responses to the PGI-S

Note: SD, standard deviation *p*-values are based on a one-way analysis of variance

The internal consistency reliability for the SF-12v2 was found to be satisfactory with the Cronbach’s alpha value of 0.848 for LPF and 0.785 for LMF.

Less than 20% of the study sample received the lowest or highest possible PCS or MCS summary scale score which was indicative of the absence of floor and ceiling effects. The minimum and maximum PCS score for the study sample was 16.95 and 67.63, respectively. The minimum and maximum MCS score was 15.75 and 68.91, respectively. The minimum and maximum PCS score for the general US population as per the SF-12v2 scoring manual is 4.92 and 69.24, respectively [8]. While the minimum and maximum MCS score for the US norm population was 8.14 and 73.24, respectively. Therefore, none of the respondents from our study sample received the lowest or highest possible score as compared to the general US population.

## Discussion

As HRQOL continues to evolve as a key endpoint among patients with hemophilia, so does the need for psychometrically-sound generic instruments which measure HRQOL. Such instruments not only allow one to ascertain the burden of hemophilia on patient HRQOL, but also compare their HRQOL to the healthy US population and across subgroups of individuals suffering from other diseases. The current study assessed the validity (factorial, convergent, discriminant, and known-groups) and internal consistency reliability of the SF-12v2, a generic measure of HRQOL, among adults with hemophilia.

Factorial validity of the SF-12v2 was tested by examining the model fit indices across three different models. A two-factor model based on the approach adopted by Maurischat and colleagues [20] was found to be the best fitting model in this population. Previous studies have also conceptualized the SF-12v2 as a two-factor model where items related to the GH, PF, RP, BP subdomains loaded onto a LPF while items related to the RE, MH, VT, SF subdomains loaded onto a LMF and the error covariance for items which belonged to the same subdomain (PF, RP, RE, and MH) were correlated. Items belonging to the same subdomain were expected to have additional commonality not explained by the latent factors due to similarities in item wording (i.e., a shared method effect), which warranted the specification of residual correlations for these items. A similar two-factor model for the SF-12v2 was found to have acceptable fit among patients with inflammatory rheumatic disease [19] and diabetes mellitus [20]. In the current study, modification indices suggested an additional residual correlation between MH09 (felt calm and peaceful) and VT10 (had a lot of energy). Residuals for these items on the SF-12 have been previously shown to be correlated by McBride et al. [39]. in a sample of diagnostic orphans (i.e., adults with a type of alcohol dependence or use disorder) and by Fleishman and Lawrence [40] in a population of non-institutionalized US civilians [39, 40].

The SF-12v2 was found to have good convergent and discriminant validity among adults with hemophilia. These findings were supported by factor loadings, the latent factor correlation, and correlations between the individual items and SF-12 subdomains. Although the latent factor correlation was high (0.83), the test for construct discriminant validity suggested that this correlation was significantly different from one. Additionally, a one-factor model had the worst model fit in the factorial validity analysis. These results provide evidence that a two-factor HRQOL model was appropriate and that the two latent factors (LPF and LMF) did indeed measure distinct concepts. This is important as a two-factor model forms the basis of the commonly reported PCS and MCS scores. Because other studies have reported have reported smaller correlations between latent physical and mental factors using the SF-12 (i.e., range 0.5–0.7) [18–20], future research should examine reasons for the higher latent factor correlation found in the current study. The high correlations between the sample-specific latent factors and the PCS and MCS scores calculated using the standard scoring approach also provide support for the use of the summary scores in HRQOL research with adults with hemophilia. Such high correlations have also been observed in studies with different populations and slightly different factor structures for the latent variables [18], providing evidence of the generalizability of the standard scoring approach for the component summaries.

The results of the current study lend support to the known-groups validity of the SF-12v2 in terms of its ability to discriminate across different symptom severity levels among adults with hemophilia. PCS and MCS means were found to be significantly different across the four symptom severity groups. Additionally, significant decreases in PCS scores were associated with increasing levels of symptom severity. Although the severe symptoms group and no symptoms group were notably different on MCS scores as expected, the overall linear trend observed with PCS scores and symptom severity was not seen in the case of MCS scores. The mean MCS for the moderate symptom severity group was slightly greater, although not statistically different, than the MCS for the mild symptom severity group.

The internal consistency reliability of the LPF and LMF summary scales was found to be good. The PCS and MCS scale scores did not indicate the presence of any floor or ceiling effects. These results may indicate that the SF-12v2 is sensitive in capturing the variation in HRQOL among adults with hemophilia.

The results of the current study must be interpreted in the light of certain limitations. The cross-sectional nature of the study precluded the assessment of the predictive validity as well as test-retest reliability of the SF-12v2. Future studies should adopt a longitudinal design in order to explore these aspects of the psychometric profile of the SF-12v2. Adults with hemophilia who participated in this study are likely to have higher physical functioning because of their ability to participate in survey research. Also, future studies must examine the measurement invariance of the SF-12v2 among adults with hemophilia in addition to testing its psychometric properties in order to ensure the appropriateness of its use in this patient population.

This was the first study to assess the psychometric properties of the SF-12v2 among adults with hemophilia. Considering that hemophilia is a rare genetic disorder, most previous published reports have employed smaller sample sizes. To the best of our knowledge, this is the first US-based study to capture the HRQOL of such a large population of adults with hemophilia. The study sample included an even distribution of patients from all regions of the country which ensures the generalizability of the study results to most adults with hemophilia in the US.

## Conclusions

This study provides evidence about the acceptable psychometric properties of the SF-12v2 among adults with hemophilia in the US. The SF-12v2 was found to be a valid and reliable generic measure of HRQOL among adults with hemophilia. The scale demonstrated adequate factorial, convergent, discriminant, and known-groups validity. The scale was found to have adequate internal consistency reliability and no evidence of floor or ceiling effects was found. Overall, the results provide basis for the future use of the SF-12v2 among adults with hemophilia and incorporating the HRQOL information obtained from these studies into health policy and clinical decision making.

## Abbreviations

CFA: Confirmatory Factor Analysis
CFI: Comparative Fit Index
GH: General Health
HRQOL: Health-related Quality of Life
HTC: Hemophilia Treatment Center
LMF: Latent Mental Factor
LPF: Latent Physical Factor
MCS: Mental Component Summary
MH: Mental Health
MI: Modification Indices
PCS: Physical Component Summary
PF: Physical Functioning
PGI-S: Patient Global Impression of Severity
RE: Role Emotional
RMSEA: Root Mean Square Error of Approximation
RP: Role Physical
SEM: Structural Equation Modeling
SF: Social Functioning
SRMR: Standardized Root Mean Square Residual
TLI: Tucker Lewis Index
VT: Vitality
WLSMV: Weighted Least Squares Minimum Variance
